# *Dictyolimon gilesii* (Plumbaginaceae), a new generic record to China, with special reference to its first plastome

**DOI:** 10.3897/BDJ.14.e177603

**Published:** 2026-03-04

**Authors:** Yuejuan Zhang, Kai Zhang, Wenzhuan Huang, Aysajan Abdusalam

**Affiliations:** 1 Xinjiang Key Laboratory of Biological Resources and Ecology of Pamirs Plateau, College of Life and Geographic Sciences, Kashi University, Kashi, China Xinjiang Key Laboratory of Biological Resources and Ecology of Pamirs Plateau, College of Life and Geographic Sciences, Kashi University Kashi China; 2 College of Life and Environmental Sciences, Hangzhou Normal University, Hangzhou, China College of Life and Environmental Sciences, Hangzhou Normal University Hangzhou China

**Keywords:** chloroplast genome, ITS, Pamir Plateau, *trn*Y-*trn*T, Xinjiang

## Abstract

**Background:**

*Dictyolimon* (Plumbaginaceae, Caryophyllales) is a small genus with three currently accepted species. Nevertheless, its distribution remains poorly known, with records confined to Afghanistan, Pakistan and India (Kashmir). During recent fieldwork on the eastern Pamir Plateau in China, we discovered a distinctive perennial herb, identifiable as *Dictyolimon gilesii*. This species is characterised by densely rosulate, coriaceous, obovate or spatulate leaves; spikelets with 3–10-flowers (typically 6), sessile; bifid petals; a pubescent calyx tube; styles free from the base; and oblique-capitate stigmas. This finding represents the first record of the genus *Dictyolimon* in China. Phylogenetic analysis, based on the ITS and *trn*Y-*trn*T loci, further support our morphology-based conclusions. Additionally, we report the first complete plastome of *Dictyolimon*, which is 152,328 bp in length and contains 121 functional genes.

**New information:**

*Dictyolimon gilesii* new to China.

## Introduction

The East Pamir Plateau (located in China) harbours unique plant resources, with 963 recorded taxa (Yang et al. 2008). Its distinctive habitat has continually attracted botanical interest. In recent years, numerous species new to China or science have been discovered in this region, such as *Astragalus dignus* Boriss (Zhai et al. 2010), *Delphinium afghanicum* Rechinger (Li et al. 2019), *Didymophysa fedtschenkoana* Regel (Yang et al. 2024), *Gagea chenghsii* J.Qiu & D.Y.Tan (Lin 2023), *Saussurea khunjerabensis* Y.S.Chen (Li et al. 2022) and *Sisymbriopsis schugnana* Botsch. & Tzvelev (Chen et al. 2019). Although these studies have significantly enhanced our understanding of the regional flora, knowledge of the plant diversity in the East Pamir Plateau remains limited, as many areas are still unexplored.

To enhance our understanding of plant diversity in the Pamir Plateau in China, we conducted a field survey in this region (Abdusalam et al. 2024). In 2024, we discovered an intriguing plant, characterised by densely rosulate, thick, coriaceous, obovate or spatulate leaves with an aculeate-mucronate apex, which we tentatively referred to as either *Limonium* or *Dictyolimon*. However, the absence of flowers prevented definitive species identification. In 2025, we observed the inflorescence of this species, which exhibited the following features: (1) spikelets loosely and subdistichously arranged along the branches, forming elongated, interrupted spicate clusters; (2) spikelets 3–10-flowered (typically 5–6), sessile; (3) a pubescent calyx tube; (4) bifid petals; and (5) styles free from the base with oblique-capitate stigmas. These morphological traits closely match the description of *Dictyolimon gilesii*. Phylogenetic analysis, based on ITS and *trn*Y-*trn*T loci, further confirmed that this species belongs to the genus *Dictyolimon*, representing the first record of this genus in China.

Plastomes are characterised by relatively small genome sizes, conservative gene content and a moderate evolutionary rate (Xu et al. 2025). Recently, plastomes have become an indispensable source of molecular data and are widely employed in studies on the taxonomy and evolution of the Plumbaginaceae (e.g. Darshetkar et al. 2021, Zhao YJ et al. 2023). Here, we report the first plastome of *Dictyolimon*, which may provide valuable insights into the evolutionary history and phylogenetic relationships of this genus.

The primary objectives of this study are to: (1) report the first occurrence of the genus *Dictyolimon* in China; (2) investigate the phylogenetic relationships of the Chinese species *Dictyolimon gilesii* and (3) present the first complete plastome of *Dictyolimon*.

## Materials and methods

### Taxon sampling

The plant material was collected from Xigelu Township in Tashekuergan Tajik Autonomous County, Kashi Prefecture, Xinjiang Uygur Autonomous Region, China. The voucher specimen (*Zhang et al. KSU-ERCP-2025-3329*) was deposited in the Herbarium of Kashi University and was utilised for phylogenetic analyses. The sampled ingroup and outgroup taxa are listed in Table 1.

### Morphological study

Species photographs and habitat were taken using a digital camera. The Deep Focus Small Specimen Digital Imaging System was employed to photograph the dissected structures. Morphometric measurements and descriptions were derived from the only flowering individual observed and more than 30 flowers from this specimen were measured and dissected.

### DNA extraction, sequencing, genome assembly and annotation

Sample preparation and DNA extraction followed protocols used in previous studies (Huang et al. 2019). High-quality genomic DNA from each sample was used for the whole genome sequencing to obtain paired-end 150 bp raw reads on the Novaseq-SE50 platform, according to the manufacturer’s procedures and about 5.2 Gb of sequences were accumulated. Raw reads with a Phred score lower than 30 were removed, retaining high-quality sequences for nuclear DNA and complete circular organelle genome assembly using the GetOrganelle v. 1.7.7.1 (Jin et al. 2020). The assembly graph viewer Bandage (Wick et al. 2015) was used to visualise the assemblies. Genomes were automatically annotated with CPGAVAS2 (Shi et al. 2019) and subsequently refined using Geneious v.11.0.3 (Kearse et al. 2012), with *Limonium sinense* (accession number: MN599096) as the reference plastome. Circular organelle genomes maps were drawn using OrganellarGenome DRAW (Lohse et al. 2013). The assembled nuclear data were aligned with published data using *Dictyolimon macrorrhabdos* as a reference (ITS accession number: LC153943) in Geneious v.11.0.3 (Kearse et al. 2012) and then annotated and extracted. The *trn*Y-*trn*T locus was extracted from the chloroplast genome. Newly-sequenced data have been deposited in the GenBase (Bu et al. 2024) in the National Genomics Data Center (CNCB-NGDC Members and Partners 2022), with accession numbers: C_AA126288.1: plastome; and C_AA126289.1: ITS).

### Phylogenetic analyses

All the sequences were aligned using MAFFT v.7.311 (Katoh and Standley 2013) and ambiguous alignment regions were trimmed using trimAl v.1.2 (Capella-Gutiérrez et al. 2009) Absent data were coded as missing. Maximum Likelihood (ML) analyses were performed in IQtree v.2.0.6 (Minh et al. 2020) with the sampling repeated 1000 times. The best-fitting substitution model (TIM3e+G4 for the ITS-partition and K3Pu+F+G4 for the *trn*Y-*trn*F-partition) was selected by ModelFinder (Chernomor et al. 2016, Kalyaanamoorthy et al. 2017), according to the Bayesian Information Criterion (BIC). For Bayesian Inference analyses, MrBayes 3.2.6 (Ronquist et al. 2012) was used to run and construct phylogenetic trees with 3,000,000 replicates, with trees sampled every 1000 generations. GTR+I+G was found to be the best-fit model for ITS-partition and GTR+G for the *trn*Y-*trn*F, according to the Akaike Information Criterion (AICc). Markov Chain Monte Carlo (MCMC) was run independently twice with one cold and three hot chains. The posterior distribution of trees was summarised by the 50% majority-rule consensus tree after discarding the first 25% of samples as burn-in.

## Data resources

### Phylogenetic results

**Figure 1.**
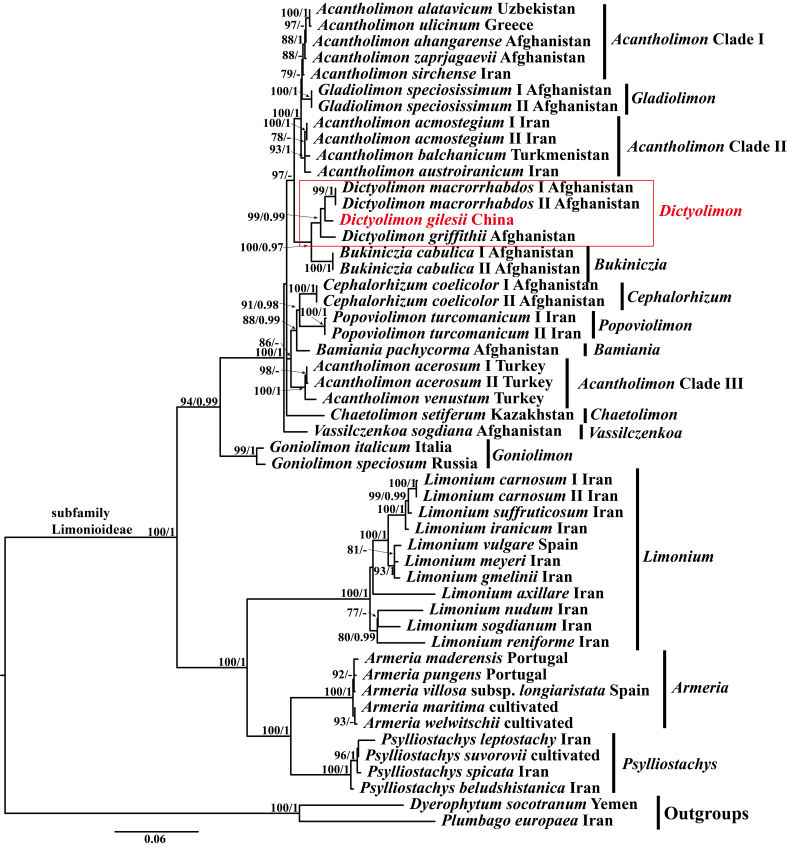
Phylogenetic tree of some Plumbaginaceae species inferred from the combined dataset (ITS and *trn*Y-*trn*T loci). The topology derived from the ML tree is shown. ML bootstrap values are shown on the left and Bayesian posterior probability values on the right. The newly-sequenced Chinese accession is coloured red.

The aligned two-loci dataset included 1,479 characters: *trn*Y-*trn*F, 867 bp; and ITS, 622 bp. Of the 14,79 aligned nucleotides, 800 were constant sites, 134 were singleton sites and 545 were parsimony-informative sites. Both ML and BI analyses generated almost identical trees with strong support for most nodes. The ML topology tree with bootstrap (BS) and posterior probability (PP) values (BS_ML_ and PP_BI_, respectively) is shown in Fig. 1.

In the phylogenetic analyses (Fig. 1), all subfamily Limonioideae species formed a well-supported clade (BS_ML_=100, PP_BI_=1). Phylogenetic results indicated that the newly-sequenced Chinese accession deeply nested within the genus *Dictyolimon* and formed a well-supported clade with two Afghani *D. macrorrhabdos* accessions and one *D. griffithii* accession (BS_ML_=99, PP_BI_=1).

### Features of plastome

**Figure 2.**
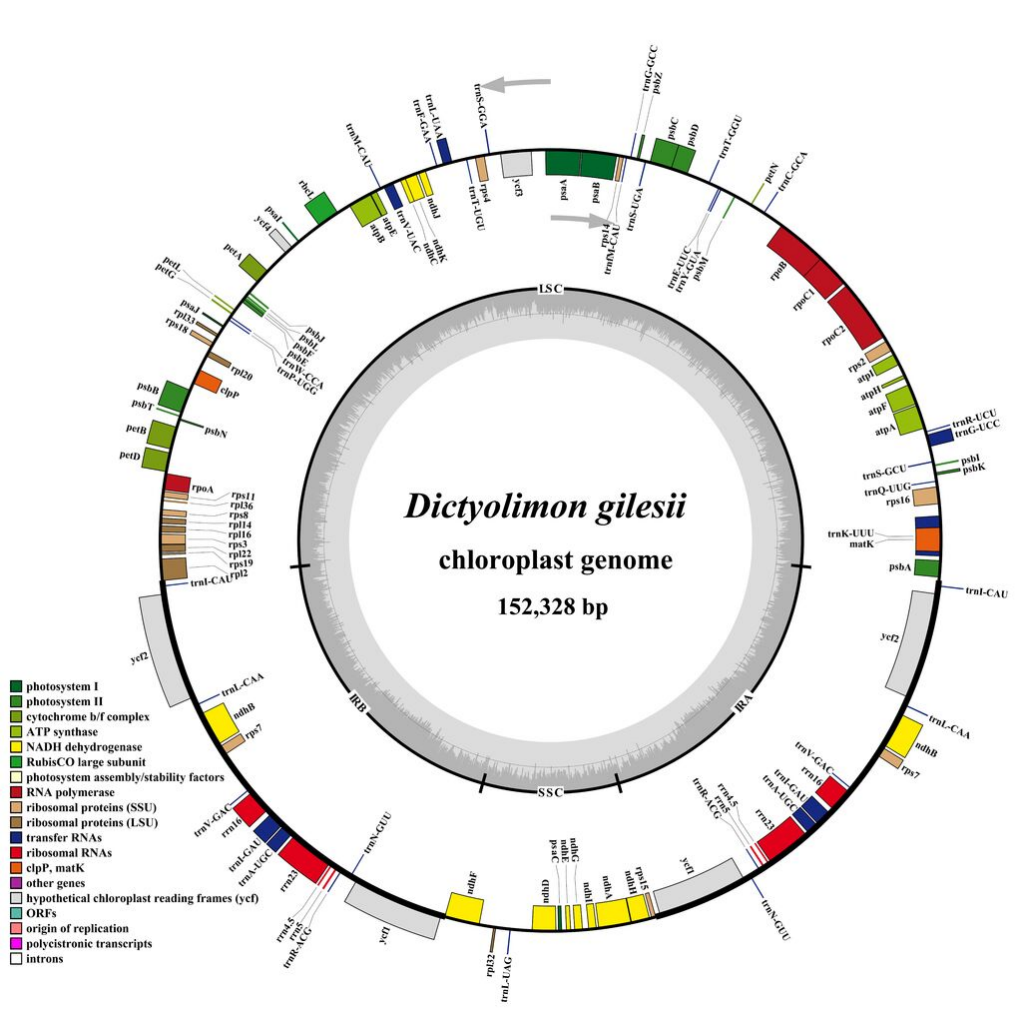
Circular genome map of the chloroplast genome of *Dictyolimon gilesii*. Genes shown on the inside of the circle are transcribed clockwise, genes on the outside are transcribed counterclockwise.

The plastome of *Dictyolimon gilesii* is 152,328 bp in length (Fig. 2). It exhibits a quadripartite structure with a large single-copy (LSC) region of 81,137 bp, a small single-copy (SSC) region of 13,221 bp and two inverted repeats (IRs) that are 28,985 bp. The plastome has a nucleotide composition of adenine 36.7% (A), 31.6% thymine (T), 16.6% cytosine (C) and 15.1% guanine (G), resulting in an overall GC content of 31.7%. The plastome contains a total of 121 functional genes, including 78 protein-coding genes, eight ribosomal RNAs (rRNA) and 35 transfer RNAs (tRNA). There are 17 intron-containing genes, with 15 genes containing one intron and both the *ycf*3 and *rps*12 genes containing two introns.

## Taxon treatments

### Dictyolimon gilesii

(Hemsl.)

E1A35AA1-5478-5C57-8120-7B137BD9EC00

**Figure 3.**
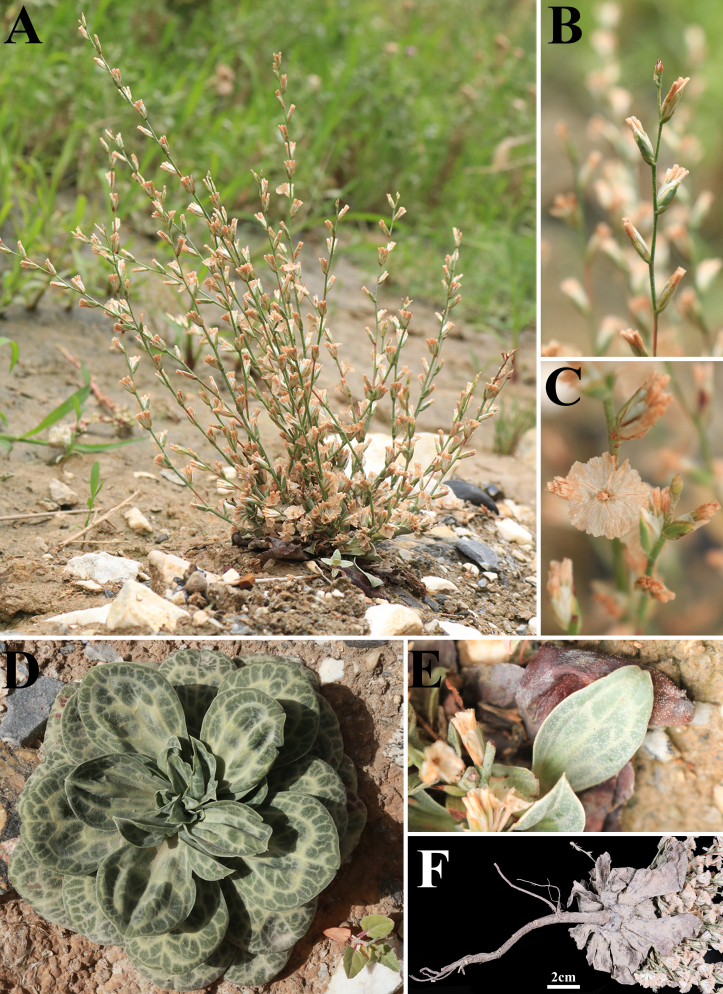
*Dictyolimon gilesii* (Hemsl.) Rech.f. **A** habitat and plant; **B** Inflorescence; **C** calyx; **D-E** leaves; **F** roots.

**Figure 4.**
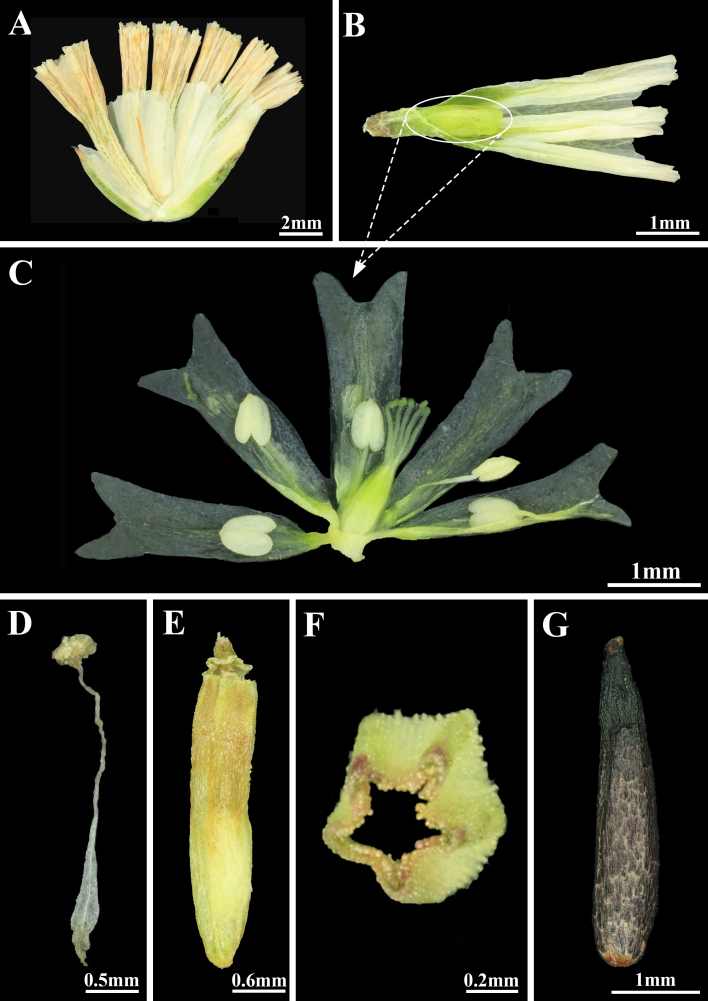
*Dictyolimon gilesii* (Hemsl.) Rech.f. **A** spikelet with six florets; **B** corolla enclosed by calyx; **C** dissected and spread-open corolla, showing the bract, pistil, stamens and petals; **D** stamen; **E** pericarp; **F** transverse section of pericarp; **G** seed.

Dictyolimon gilesii (Hemsl.) Rech.f., Flora Iranica 108: 22. 1974 (Figs. 3–4)
≡ *Statice gilesii* Hemsl., Hooker's Icones Plantarum.18: tab. 1737. 1888
HOLOTYPE: Hab. Shoghot, at 1828 to 2133 m elev., south of Hindu Kush; Gilgit Expendition, Dr. Giles.(K [digital image!]).

#### Materials

- **Type status:** Other material. **Occurrence:** catalogNumber: *KSU-ERCP-2025-3329*; occurrenceRemarks: Herbarium of Kashi University; recordedBy: Yuejuan Zhang; Kai Zhang; Mierkamili Maimaiti; **Location:** country: China; stateProvince: Xinjiang Uygur Autonomous Region; county: Kashi Prefecture, Tashekuergan Tajik Autonomous County; municipality: Kekexiluge Township; verbatimElevation: 2472 m; verbatimLatitude: 37°50′14.04″ N; verbatimLongitude: 75°42′22.18″ E; **Event:** verbatimEventDate: 26 July 2025

#### Description

Perennial herb, ca. 25 cm tall, glabrous, glaucous, minutely lepidote. Tap-rooted. Leaves densely rosulate, thick, coriaceous, obovate or spatulate, ca. 2.5–6.3 cm long, apex aculeate-mucronate. Scape solitary, flexuous, usually simple, sometimes with 2–3 short lateral branchlets, with flowers at or below the middle. Spikelets loosely subdistichously arranged along the branches, forming long interrupted spicate clusters. Spikelets 3–10-flowered (usually 5–6-flowered), sessile. Bracts and bracteoles similar, broad and short, almost entirely scarious-hyaline; outer bract shorter. Calyx shortly 10-lobed, tube strongly 10-ribbed, pubescent. Petals 5, bifid. Corolla persistent after anthesis. Stamens 5 opposite the corolla lobes. Filaments linear, widened in the lower part. Ovary glabrous. Styles free from the base, stigmas oblique-capitate. Pericarp dry, membranous, 5–striate, apex easily deciduous. One seed in each calyx tube, seed coat dark brown to black, longitudinally rugose-sulcate (Figs 3, 4).

#### Distribution

*Dictyolimon gilesii* is previously known only from Pakistan (Hooker 1888) and Afghanistan (https://records.data.kew.org/occurrences/8a8ba91b-429b-4b84-8d10-069781faaa12?lang=zh-CN). In China, this species is known only from the eastern Pamir Plateau, Xinjiang Uygur Autonomous Region.

## Discussion

*Dictyolimon gilesii* is characterised by the following combination of morphological features: (1) a perennial herbaceous habit (Fig. 3A; Hooker (1888)); (2) leaves densely rosulate, thick, coriaceous, obovate or spatulate, 2.5–6.3 cm long, with an aculeate-mucronate apex (Fig. 3D and E; Hooker (1888)); (3) spikelets 3–10-flowered (typically 6) and sessile (Fig. 4A; Hooker (1888)); (4) bifid petals, styles free from the base and oblique-capitate stigmas (Fig. 4C; Rechinger and Schiman-Czeika (1974)); (5) a glabrous ovary (Fig. 4C; Hooker (1888)); and (6) a pubescent calyx tube (Fig. 4A; Hooker (1888)).

Plumbaginaceae Juss., a nearly cosmopolitan family in the order Caryophyllales, exhibits its greatest species diversity in the Northern Hemisphere (Malekmohammadi et al. 2024), particularly across the Mediterranean and Irano-Turanian Regions, as well as in southern Africa, southern South America and western Australia (Hernández-Ledesma et al. 2015). The family is predominantly composed of halophytes and psammophytes that inhabit saline or coastal environments, along with a substantial clade of cold-adapted orophytes restricted to arid montane regions (Malekmohammadi et al. 2024). *Dictyolimon* belongs to a clade that is distinctly characterised by its non-halophytic ecology (Caperta et al. 2020). We discovered this species at the base of high mountains in a perennially arid region, which represents the only known distribution area of *Dictyolimon* in China.

The genus *Dictyolimon* currently comprises three accepted species worldwide: *D. gilesii*, *D. griffithii* (Aitch. & Hemsl.) Rech.f. and *D. macrorrhabdos* (Boiss.) Rech.f. While *D. gilesii* and *D. griffithii* are often difficult to distinguish, a primary diagnostic difference lies in the number of flowers per spikelet: *D. gilesii* typically bears 6-flowered spikelets (Fig. 4A; Hooker (1888)), whereas those of *D. griffithii* bears only one to two flowers (Rechinger and Schiman-Czeika 1974). The tree topology supports the classification of the *D. accession* from Xinjiang Province within *D. macrorrhabdos* (Fig. 1). Morphologically, *D. gilesii* can be easily confused with *D. macrorrhabdos*. However, it can be distinguished by the following characteristics: (1) *D. gilesii* has spikelets with typically six flowers (Fig. 4A; Koie and Rechinger (1963)), compared to the 3–5 flowers in *D. macrorrhabdos* (Rechinger and Schiman-Czeika 1974); and (2) the calyx tube is pubescent in *D. gilesii* (Fig. 4A; Hooker (1888)), but the calyx is glabrous or sparsely papillate along the ribs in *D. macrorrhabdos* (Rechinger and Schiman-Czeika 1974).

*Dictyolimon* is morphologically closest to *Bukiniczia* （Moharrek et al. 2017). Notably, our results revealed that these two genera form sister groups (Fig. 1), consistent with previous phylogenetic studies (Moharrek et al. 2017; Koutroumpa et al. 2018). Interestingly, Moharrek et al. (2017) proposed merging eight genera, including *Dictyolimon*, into *Acantholimon*, suggesting a broad circumscription of *Acantholimon* due to the non-monophyly of the species within this genus. Furthermore, our results demonstrate that *Acantholimon* is divided into three clades (Fig. 1), underscoring the need for detailed phylogenetic and taxonomic studies within the *Acantholimon* *s.l.*

## Supplementary Material

XML Treatment for Dictyolimon gilesii

## Figures and Tables

**Table 1.** Voucher information and GenBank accession numbers of ITS and *trn*Y-*trn*T sequences used in this study; new sequences in bold. “—” means data missing.

**Species**	**Location**	**Vouchers (herbarium code)**	***nr* DNA ITS**	***trnY-T***
*Acantholimon acerosum* (Willd.) Boiss.	Turkey	*Eren s.n.* (W 2014-12433)	LC153805	LC153954
*Acantholimon acerosum* (Willd.) Boiss.	Turkey	*Aedo et al. 6249* (MA 688508)	LC153806	LC153955
*Acantholimon acmostegium* Boiss. & Buhse	Iran	*Kazempour-Osaloo & Moharrek s.n.* (TMUH 92351)	LC153807	LC153956
*Acantholimon acmostegium* Boiss. & Buhse	Iran	*Assadi 84464* (TARI)	AB979532	AB979600
*Acantholimon ahangarense* Rech.f. & Schiman-Czeika	Afghanistan	*Rechinger 18860* (W 1975-0013927)	LC153808	LC153957
*Acantholimon alatavicum* Bunge	Uzbekistan	*Vašák s.n.* (MA 642281)	LC153809	LC153958
*Acantholimon austroiranicum* Rech.f. & Schiman-Czeika	Iran	*Mirtadzadini 83216* (TARI)	AB979538	AB979605
*Acantholimon balchanicum* Korovin	Turkmenistan	*Nikitin s.n.* (W 1985-0007275)	LC153816	LC153965
*Acantholimon sirchense* Assadi & Mirtadz.	Iran	*Mirtadzadini s.n.* (SHBU 1266)	AB979581	AB979640
*Acantholimon ulicinum* (Willd. ex Schult.) Boiss.	Greece	*Merxmüller & Podlech 30886* (M 0276189)	LC153920	LC154036
*Acantholimon venustum* Boiss.	Turkey	*Aldasoro et al. 2724* (MA 689899)	LC153925	LC154040
*Acantholimon zaprjagaevii* Lincz.	Afghanistan	*Podlech 16364* (M 0276224)	LC153931	LC154045
*Armeria maderensis* Lowe	Portugal	*Piñeiro 112RP10* (MA)	LC153932	LC154046
*Armeria maritima* (Mill.) Willd.	Iran	Cultivated National Botanical Garden Iran	AB979588	AB979647
*Armeria pungens* (Brot.) Hoffmanns. & Link	Portugal	*Nieto Feliner 4457GN* (MA)	LC153933	LC154047
*Armeria villosa subsp. longiaristata* (Boiss. & Reut.) Nieto Fel.	Spain	*Nieto Feliner 4255GN* (MA)	LC153934	LC154048
*Armeria welwitschii* Boiss.	Iran	Cultivated National Botanical Garden Iran	AB979589	AB979648
*Bamiania pachycorma* (Rech.f.) Lincz.	Afghanistan	*Hedge & Wendelbo 4103* (E)	LC153935	LC154049
*Bukiniczia cabulica* (Boiss.) Lincz.	Afghanistan	*Podlech 21781* (M 0276239)	LC153936	LC154050
*Bukiniczia cabulica* (Boiss.) Lincz.	Afghanistan	*Alpay s.n.* (MSB 006138)	LC153937	LC154051
*Cephalorhizum coelicolor* (Rech.f.) Rech.f.	Afghanistan	*Anders 9314* (MSB 006131)	LC153938	LC154052
*Cephalorhizum coelicolor* (Rech.f.) Rech.f.	Afghanistan	*Podlech 11313* (MSB 006132)	LC153939	LC154053
*Chaetolimon setiferum* (Bunge) Lincz.	Kazakhstan	*Neustruyeva et al. s.n.* (M)	LC153940	——
*Dictyolimon griffithii* (Aitch. & Hemsl.) Rech.f.	Afghanistan	*Podlech 16138* (MSB 006130)	LC153941	——
*Dictyolimon macrorrhabdos* (Boiss.) Rech.f.	Afghanistan	*Podlech 30260* (MSB 006135)	LC153942	LC154054
*Dictyolimon macrorrhabdos* (Boiss.) Rech.f.	Afghanistan	*Podlech 18032* (MSB 006134)	LC153943	LC154055
*Dyerophytum socotranum* (Balf.f.) J.R.Edm., M.Malekm. & Koutr.	Yemen	*Miller et al. 10300* (E 0035670)	LC153944	LC154056
*Gladiolimon speciosissimum* (Aitch. & Hemsl.) Mobayen	Afghanistan	*Podlech 16945* (MSB 006125)	LC153945	LC154057
*Gladiolimon speciosissimum* (Aitch. & Hemsl.) Mobayen	Afghanistan	*Amin 74* (MSB 006126)	LC153946	LC154058
*Goniolimon italicum* Tammaro, Pignatti & Frizzi	Italia	*Conti s.n*.	LC217873	——
*Goniolimon speciosum* (L.) Boiss.	Russia	*Castroviejo 14315SC* (MA 614247)	LC217874	——
*Limonium axillare* (Forssk.) Kuntze	Iran	*Maassoumi & Abu Hamzeh 52016* (TARI)	LC153947	LC154059
*Limonium carnosum* (Boiss.) Kuntze	Iran	*Assadi 79082* (TARI)	LC153948	LC154060
*Limonium carnosum* (Boiss.) Kuntze	Iran	*Kazempour-Osaloo s.n.* (TMUH 92360)	LC153949	LC154061
*Limonium gmelinii* (Willd.) Kuntze	Iran	*Memariani & Zanguei 41461* (FUMH)	AB979591	AB979650
*Limonium iranicum* (Bornm.) Lincz.	Iran	*Memariani & Akhani 39323* (FUMH)	AB979592	AB979651
*Limonium meyeri* (Boiss.) Kuntze	Iran	*Kazempour-Osaloo s.n.* (TMUH 89213)	AB979593	AB979652
*Limonium nudum* (Boiss. & Buhse) Kuntze	Iran	*Assadi & Maassoumi 21019* (TARI)	LC153950	LC154062
*Limonium reniforme* (Girard) Lincz.	Iran	*Joharchi & Zanguei 1484* (FUMH)	AB979594	AB979653
*Limonium sogdianum* (Popov) Ikonn.-Gal.	Iran	*Joharchi 34168* (FUMH)	AB979595	AB979654
*Limonium suffruticosum* (L.) Kuntze	Iran	*Joharchi 35188* (FUMH)	LC153952	AB979655
*Plumbago europaea* L.	Iran	*Zanguei 38466* (FUMH)	AB979599	AB979659
*Popoviolimon turcomanicum* (Popov) Lincz.	Iran	*Joharchi & Zanguei 36270* (FUMH)	AB979590	AB979649
*Popoviolimon turcomanicum* (Popov) Lincz.	Iran	*Faghihnia & Zanguei 28978* (FUMH)	JX983658	——
*Psylliostachys beludshistanica* Roshk.	Iran	*Faghihnia & Zanguei 18122* (FUMH)	AB979596	AB979656
*Psylliostachys leptostachya* (Boiss.) Roshk.	Iran	*Joharchi & Zanguei 14525* (FUMH)	AB979597	AB979657
*Psylliostachys spicata* (Willd.) Nevski	Iran	*Zanguei 34446* (FUMH)	AB979598	AB979658
*Psylliostachys* suvorovii (Regel) Roshk.	Austria	Cultivated Insbruck Botanical Garden	AJ132446	——
*Vassilczenkoa sogdiana* (Lincz.) Lincz.	Afghanistan	*Podlech 21130* (M 0276244)	——	LC154063
*Limonium vulgare* Hill.	Spain	*Roselló JAR 96085*	AJ222839	——
